# Protein A immunoadsorption combination with immunosuppressive therapy improves neuropsychiatric systemic lupus erythematosus: A case report

**DOI:** 10.1002/ccr3.3140

**Published:** 2020-07-16

**Authors:** Feng Wen, Xiaoling Wu, Ruyi Liao, Zhonglin Feng, Zhilian Li, Xia Fu, Lei Fu, Sijia Li, Zhuo Li, Sheng Li, Wenjian Wang, Biao Huang, Chaosheng He, Shi Wei, Xinling Liang, Shuangxin Liu

**Affiliations:** ^1^ Department of Nephrology Guangdong Provincial People's Hospital Guangdong Academy of Medical Sciences Guangzhou China; ^2^ Medical School South China University of Technology Guangzhou China; ^3^ Department of Radiology Guangdong Provincial People's Hospital Guangdong Academy of Medical Sciences Guangzhou China

**Keywords:** neuropsychiatric systemic lupus erythematosus, Protein A immunoadsorption

## Abstract

We described protein A immunoadsorption combination with immunosuppressive treatment improved rapidly a patient with Neuropsychiatric systemic lupus erythematosus.

## Graphical abstract

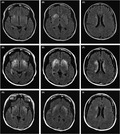

## 1 INTRODUCTION

Neuropsychiatric systemic lupus erythematosus (NPSLE) is usually associated with a poor prognosis. However, there are few effective treatments for NPSLE. We described protein A immunoadsorption treatment for a patient with NPSLE. The patient's brain lesions were improved rapidly after two sessions of protein A immunoadsorption combination with immunosuppressive agent treatment.

Systemic lupus erythematosus (SLE) is a chronic autoimmune disease with a broad spectrum of clinical presentations.^1^ The neuropsychiatric SLE (NPSLE) presents with heterogeneous symptoms, such as headache, cognitive impairment, memory loss, seizure, and stroke, which contributes considerably to morbidity and mortality. The development and deterioration of NPSLE are associated with the formation of a large number of autoantibodies and circulating immune complexes in the body, which could cause severe brain damage and even life‐threatening.^2^ Inhibiting or removing pathogenic autoantibodies could prevent their pathological outcomes.^1^ Severe NPSLE should be treated with immunosuppressive and biological agents. However, some patients are not sensitive to these drugs. Protein A immunoadsorption (IAS) has been shown to improve disease activity and lower glucocorticoid dosages in lupus nephritis.^3^ Braun and colleagues reported that protein A immunoadsorption had an effect on severe and therapy‐resistant SLE, which suggested IAS as a possible option when other therapies were ineffective.^4^ We presented a case of NPSLE, who received three sessions of IAS treatment after poor responses to immunosuppressive agents. The significant improvement of brain magnetic resonance imaging (MRI) manifestations was observed after the comprehensive treatments.

## 2 CASE HISTORY

**FIGURE 1:**
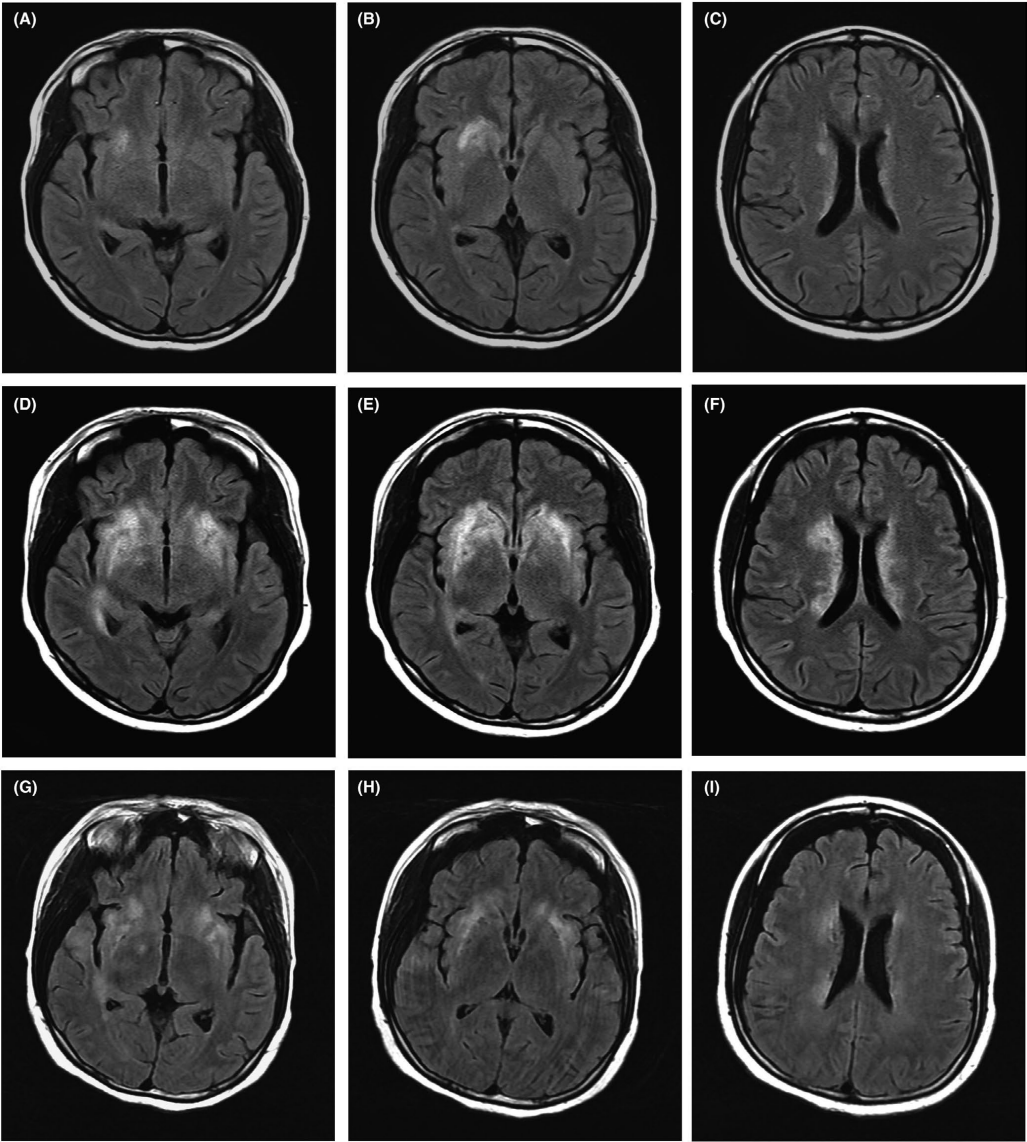
The changes in brain lesions in patient's MR scan during treatment. The abnormal signals could be found in axial fluid‐attenuated inversion recovery (FLAIR) sequences. Patients' first MRI showed diffuse and symmetric hyperintense areas in the bilateral periventricular white matter, basal ganglia, and insula (A‐C). Compared with first MR scan, the area of brain lesions expanded obviously before protein A immunoadsorption treatment (D‐F). After two sessions of protein A immunoadsorptions, FLAIR images of MRI decreased significantly area of lesions (G‐I).

A 20‐year‐old woman, who had fever and red rash on her face, was admitted firstly in April 2016. Plasma antinuclear antibody (ANA), anti‐dsDNA, and proteinuria were increased. She was diagnosed as SLE and lupus nephropathy (type Ⅱ) by kidney biopsy. Oral methylprednisolone (MP, 40 mg, daily) and hydroxychloroquine (HCQ, 100 mg, twice daily) were prescribed. The rash faded, and the proteinuria completely relieved after 2 months' treatments. She regularly decreased the dosage of MP. In August 2018, when the MP was reduced to 4mg daily, the rash on face reappeared. Two months later, her right eye presented with blurred vision. The blood tests indicated an elevation of ANA titer to 1:1280 and anti‐dsDNA concentration to 74.5 IU/mL. The blood IgG concentration was 18.1 g/L. The plasma C3 level slightly declined, and proteinuria stayed normal. HCQ was stopped immediately, and MP of 12 mg daily was given. Although the rash soon improved, the patient had fever, and the visual impairment developed on both sides. The first cerebrospinal fluid (CSF) examination showed a normal pressure of 170 mmH_2_O, with an increased protein concentration of 922 mg/L, and a slight elevation of IgG level and leukocyte count. Pathogen examinations including bacterial and fungal cultures and variety of viral antibodies were all negative in the CSF and blood tests. In brain MRI scan, the axial fluid‐attenuated inversion recovery (FLAIR) sequence showed hyperintense in the right basal ganglia and bilateral periventricular white matter, without abnormal enhancement (Figure 1A‐C). Ophthalmic examinations suggested retinal vasculitis in both eyes. The patient was identified as NPSLE with a disease activity score of 19 (brain damage, visual impairment, rash, and fever). Intravenous MP (500 mg daily for 3 days) and cyclophosphamide (200 mg every other day) were prescribed. Prednisolone of 45 mg daily was given subsequently.

However, the vision did not improve after intravenous MP combined with cyclophosphamide treatment. The second brain MRI scan showed that lesions of abnormal hyperintense were increased in the right lateral temporal cortex, right thalamus bilateral basal ganglia, insular lobe external capsule, and periventricular white matter on FLAIR images. Intravenous MP (500 mg daily for 3 days) was used again, and rituximab (500 mg) was prescribed twice. Intrathecal injections of methotrexate and dexamethasone were performed weekly for a total of six times. Although the patient was treated with intensive therapy, the CSF examinations turned out to be a significant increase in pressure (218 mmH_2_O) and protein concentration (2187 mg/L), and area of brain lesions still expanded by MRI scan (Figure 1D‐F). The patient's consciousness becomes worse accompanied by dysphasia.

Because prednisolone and immunosuppressive agents had no effects on retinal vasculitis and consciousness, protein A IAS (Koncen Biotech. Co., Ltd.) was performed. Three protein A IAS sessions were executed. Each session ran six cycles, and total volume of plasma adsorption was 3600 mL. Her consciousness improved soon after the first session. After the second session, the examination of CSF showed a normal pressure of 165 mmH_2_O and a decrease in protein concentration to 547 mg/L. The brain lesions on MRI were found to be significantly reduced (Figure 1G‐I). Plasma IgG decreased significantly after each IAS sessions (Table 1). The protein A IAS was interrupted because of catheter‐related deep vein thrombosis after the third section. Two weeks later, the patient discharged with oral MP (40 mg daily). Her consciousness and strength completely recovered after 1 month, but the vision has still not restored.

**TABLE 1:** Changes in blood immune indicators before and after immunoadsorption sessions

Sections	Session 1		Sessions 2		Sessions 3	
	Pre	Post	Pre	Post	Pre	Post
IgG (g/L)	9.52	2.74	4.36	0.33	6.07	1.19
IgA (g/L)	1.54	1.25	1.28	0.93	1.11	0.90
IgM (g/L)	0.32	0.23	0.22	0.16	0.16	0.14
κ (g/L)	2.65	0.92	1.30	<0.40	1.69	0.45
λ (g/L)	1.05	0.40	0.55	0.21	0.66	0.23
C3 (mg/L)	1081	855	1014	835	1189	1076
C4 (mg/L)	251	248	286	242	336	303
Leukocyte (×10^9^/L)	10.67	20.52	7.93	7.55	5.31	7.24
Platelet (×10^9^/L)	213	126	103	66	151	104

## 3 DISCUSSION

Glucocorticoid combination with intravenous pulse cyclophosphamide is the most classical strategy for SLE with major organs involved.^5^ It is effective in many cases, but not all situations. When common strategies are contraindicated or failed in life‐threatened SLE, extracorporeal treatments are performed. Although plasma exchange could nonselectively remove the antibodies, it is reported to be not effective in prospective trial.^6^ In contrast, IAS can reduce significantly the concentration of IgGs in blood without loss of coagulation factors and plasma albumin. Stummvoll and colleagues found that IAS improved significantly disease activity and lowered glucocorticoid dosages in SLE patients.^3^, ^7^ These patients showed benefits within 3 months of treatment and stabilization thereafter. IAS might be used as an optional treatment when other therapies are not effective in SLE.^4^

In this case, the patient's condition deteriorated during the process of glucocorticoid reduction, and her brain was involved in damage. Although the changes in laboratory tests were mild, the neuropsychiatric symptoms and brain MRI were progressive. The symptoms became worse, even if intensive immunosuppressive and biological agents were prescribed. It was worthy of note that the brain MRI was rapidly improved after two sessions of IAS were performed, together with intravenous cyclophosphamide and immunosuppressive therapy.

The ability of elimination of circulating complexes by protein A IAS was confirmed in several autoimmune diseases.^8^, ^9^, ^10^ Braun and colleagues reported the elimination kinetics of IgGs and circulating immune complexes in vivo during the treatment of severe SLE.^11^ It showed that all IgG subclasses were removed from the patient's plasma. The half‐time of IgG elimination is 4.8 days during intermittent therapy. Protein A IAS decreases IgG levels in the blood, leads to a reduction in IgGs in cerebrospinal fluid, and then improves the clinical symptoms of NPSLE. The immunosuppressive treatment could reduce the recomposition and redistribution. Thus, the treatment of adsorption could be better, when immunosuppressive agents are prescribed. Because of competition for protein A–binding sites, intravenous immunoglobulin prevents the rapid decline of IgG in the patients, which is suggested to be avoided during protein A IAS treatment.^11^

Neuropsychiatric systemic lupus erythematosus is associated with a worse prognosis, and brain MRI scan is recommended to evaluate brain lesions inpatients.^12^ The most frequent MRI findings are focal lesions in subcortical white matter, cortical atrophy, diffuse cortical gray matter changes, and less brain and corpus callosum volumes in patients with NPSLE.^13^, ^14^ The patient showed abnormal hyperintense in right basal ganglia at the beginning, similar to cerebral infarction. When the disease progressed, atrophy of the brain, diffuse white matter, and multiple nuclear lesions were identified. The formation of these manifestations contributes to blood‐brain barrier dysfunction, cerebrovascular disease, serum, and CSF autoantibody–mediated injury.^15^ Microinfarcts may be responsible to retinal vasculitis and basal ganglia lesions in this patient. The autoantibody deposition in the brain plays an important role in the pathogenesis.^16^ Some of them, such as antiphospholipid antibodies, are reported more likely to be elevated in NPSLE.^17^ Besides, evidences suggest that these immunoglobulins and immune cells could leak into brain tissue and cause immune responses.

## 4 CONCLUSION

In summary, we firstly reported a case of NPSLE with severe brain damage, which was improved rapidly after protein A IAS combination with immunosuppressive treatment. Although IAS treatment is effective in the patient with NPSLE, we need to do more studies to verify the effectiveness of protein A immunoadsorption in future.

## CONFLICT OF INTEREST

None declared.

## AUTHOR CONTRIBUTIONS

All authors: read and approved the manuscript. FW and RYL: collected the clinical data of the patient, performed the literature search, and drafted the manuscript. XLW: provided and evaluated the MR image and drafted part of the manuscript. ZLF and ZLL: participated in the data collection. XF, SJL, ZL, SL, WJW, CSH, and WS: established the diagnosis and agreed the patient's management plan. BH: participated in the preparation the results of the MR images. XLL and SXL: supervised and drafted the manuscript.

## ETHICAL APPROVAL AND CONSENT TO PARTICIPATE

Because it is a case report, this study was exempted from the research ethics committee review (Ethics committee of Guangdong Provincial People's Hospital). The patient has given consent for publication.

## CONSENT FOR PUBLICATION

Written informed consent was obtained from the patient for publication of this case report. A copy of the consent form is available for review and can be provided on request.

## Data Availability

The clinical data regarding this case are stored in hospital medical records.
